# Supplementary material for: Metabolic targeting of cancer associated fibroblasts overcomes T-cell exclusion and chemoresistance in soft-tissue sarcomas
Source: Nat Commun. 2024 Mar 20;15:2498. doi: 10.1038/s41467-024-46504-4 (PMC10954767; doi:10.1038/s41467-024-46504-4)
Supplement: Supplementary file 1 — Supplementary Information [file 41467_2024_46504_MOESM1_ESM.pdf]

SUPPLEMENTARY INFORMATION

FOR

Metabolic targeting of cancer associated fibroblasts overcomes T-cell exclusion and chemoresistance in soft-tissue sarcomas

Marina T. Broz[1], Emily Y. Ko[2], Kristin Ishaya[2], Jinfen Xiao[2], Marco De Simone[2], Xen Ping Hoi[1], Roberta Piras[1], Basia Gala[1], Fernando H. G. Tessaro[2], Anja Karlstaedt[1,3,4], Sandra Orsulic[4,5], Amanda W. Lund[6], Keith Syson Chan[7], & Jlenia Guarnerio[1,2,4,8].

Corresponding Author: Jlenia Guarnerio

Email: jlenia.guarnerio@cshs.org

Broz et al. Supplementary Fig. 1

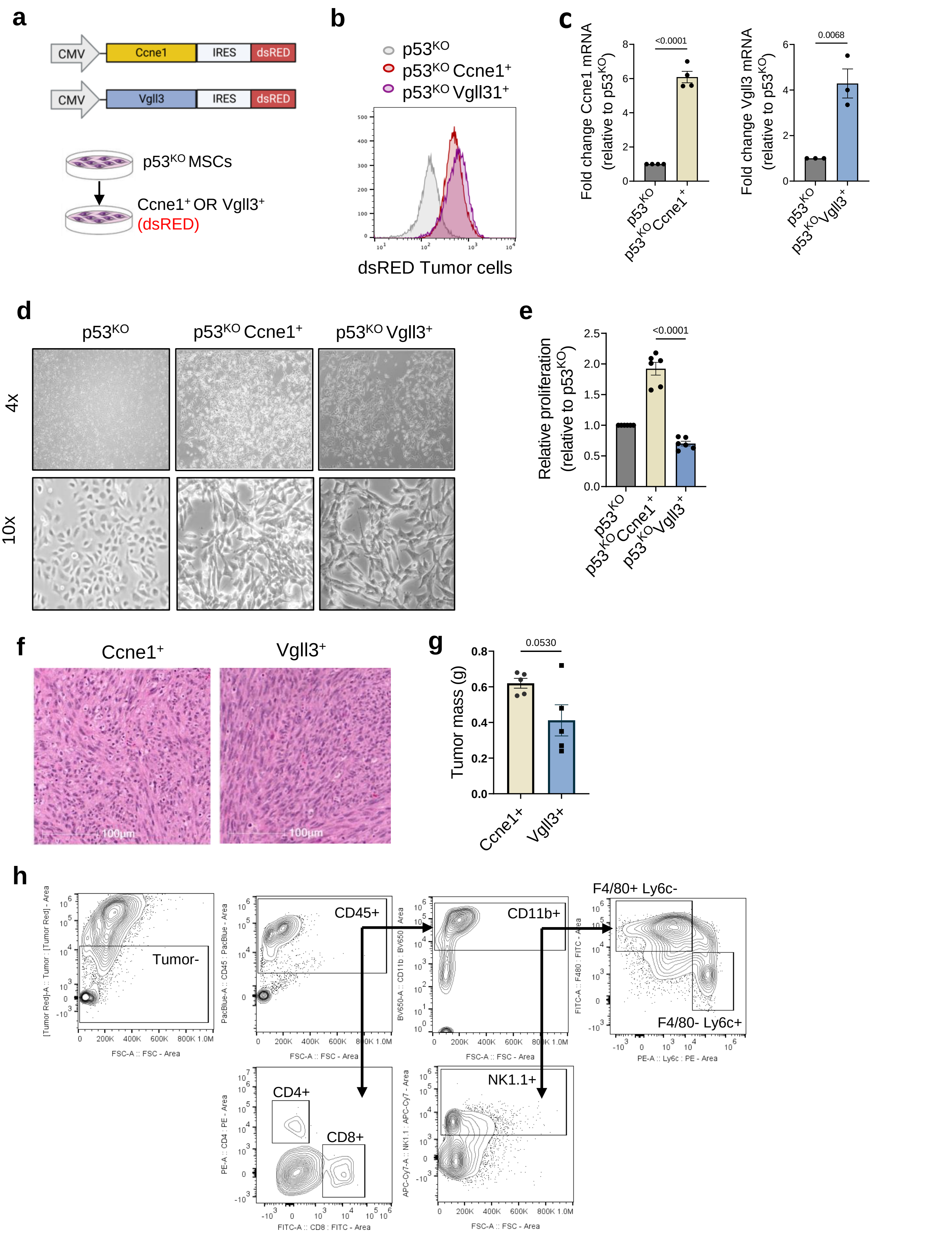

Supplementary Fig. 1: Validation of immunocompetent murine sarcoma models

a Schematic of *Ccne1* and *Vgll3* retroviral expression vectors. **b** Flow cytometry expression of dsRED in the resulting cell lines **c** qRT-PCR gene expression of *Ccne1* (left) in non-transformed $p53^{KO}$ MSC and transformed $p53^{KO}$ Ccne1+ cells and *Vgll3* (right) in $p53^{KO}$Vgll3+ cells (n=4 biological replicates for $p53^{KO}$ Ccne1+, n=3 biological replicates for $p53^{KO}$Vgll3+). **d** Morphology of $p53^{KO}$, $p53^{KO}$ $Ccne1^{+}$, and $p53^{KO}$ $Vgll3^{+}$ cell lines at 4x and 10x magnification. Images from one experiment with n=3 are shown, data representative of three independent experiments. **e** Crystal violet absorbance values (595nm) were used to quantify the relative proliferation of $p53^{KO}$ $Ccne1^{+}$ and $p53^{KO}$ $Vgll3^{+}$ relative to $p53^{KO.}$ Data from one experiment with n=6 are shown, data representative of three independent experiments. **f** H&E staining of tissue sections from $Ccne1^{+}$ or $Vgll3^{+}$ tumors. **g** Tumor mass of the resulting Ccne1+ and Vgll3+ tumors (n=5 mice). **h** Relative flow cytometry gating strategy for immune cells corresponding to data in Fig. 1c. Source data are provided as a Source Data file.

Broz et al. Supplementary Fig. 2

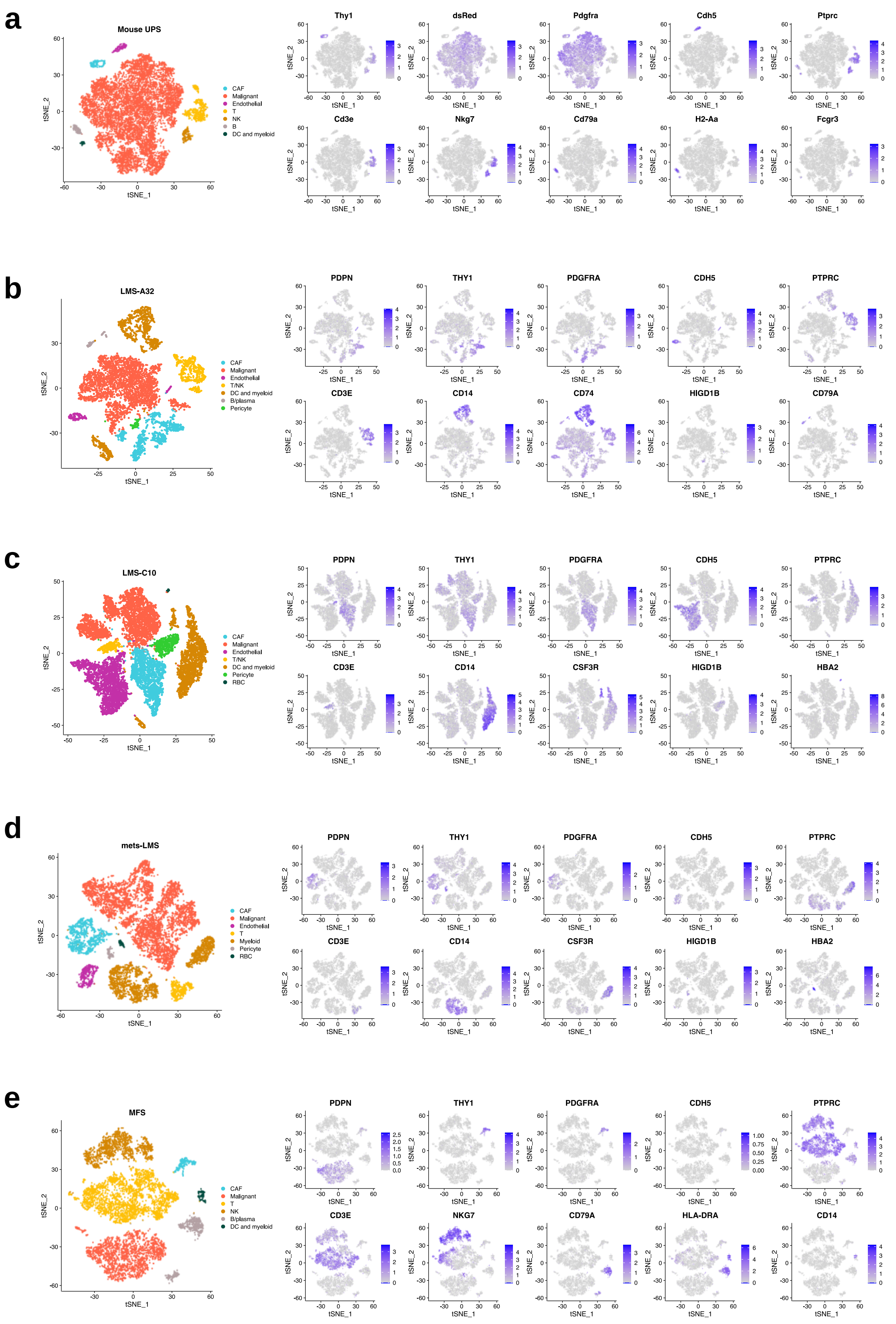

Supplementary Fig. 2: Cell-cluster identifications of murine and human sarcomas

a t-SNE of major cell types identified in mouse UPS (Ccne1+ immune excluded model) (left) and markers used to determine cluster identity (right). **b-c** t-SNE of major cell types identified in human primary leiomyosarcoma (left) and markers used to determine cluster identity (right). **d** t-SNE of major cell types identified in human metastatic leiomyosarcoma (left) and markers used to determine cluster identity (right). **e** t-SNE of major cell types identified in human primary myxofibrosarcoma (left) and markers used to determine cluster identity (right).

Broz et al. Supplementary Fig. 3

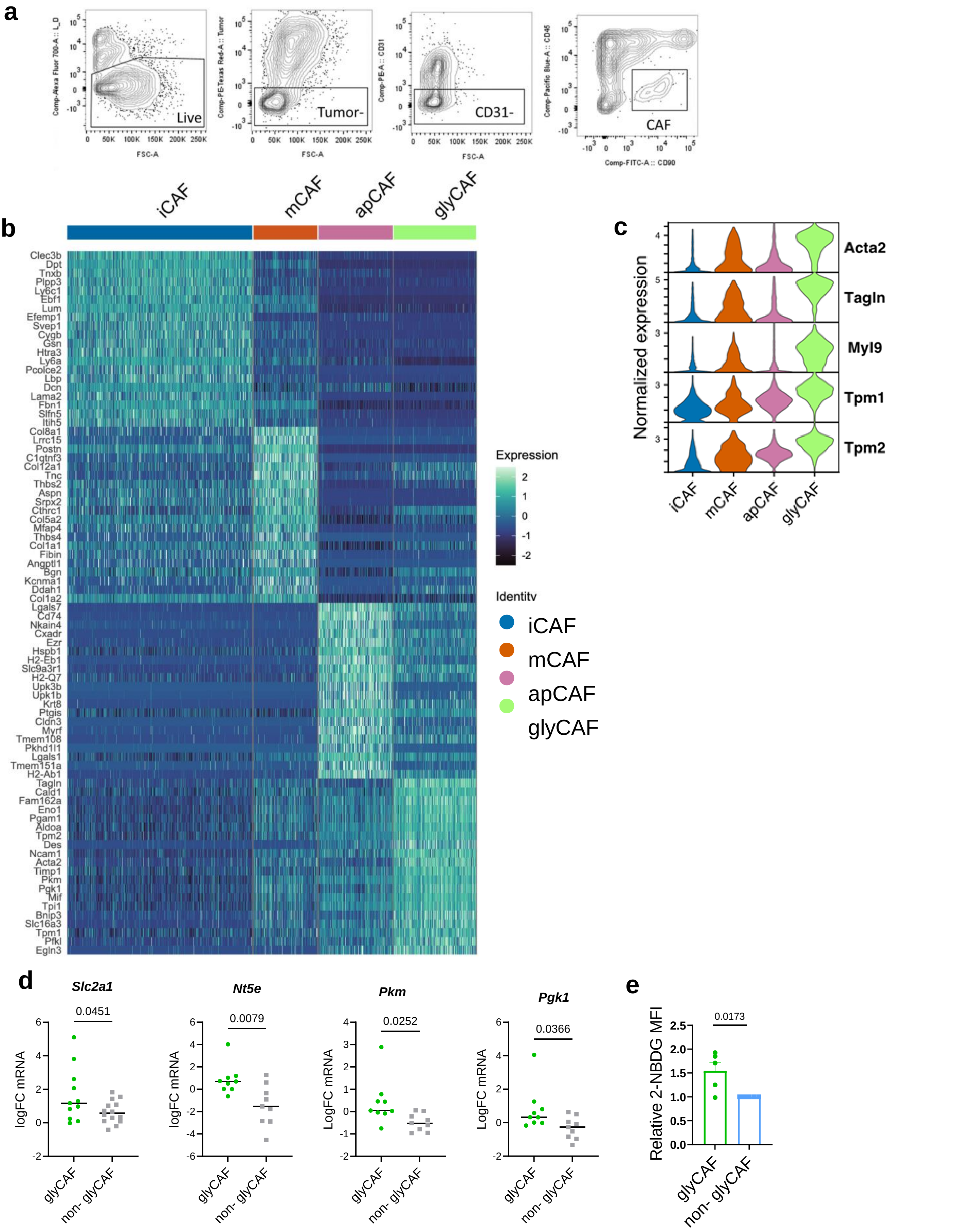

Supplementary Fig. 3: Profiling of murine sarcoma CAF by scRNA-seq

a Flow cytometry gating strategy used to isolate CAF from mouse UPS tumors by gating on live, dsRED$^-$ (Tumor) CD45$^-$ CD31$^-$ CD90$^+$ cells. **b** Expression of top marker genes differentially expressed by the CAF clusters. **c** Expression of classical myCAF markers across the fibroblast clusters. **d** qRT-PCR expression of *Slc2a1, Nt5e, Pkm1*, and *Pgk1* in FACS-sorted glyCAFs (CD90$^+$ CD73$^+$) and non-glyCAF (CD90$^+$ CD73$^-$) isolated from Ccne1+ tumors (*Slc2a1*: glyCAF n=11 mice, non-glyCAF n=14 mice. *Nt5e, Pkm, Pgk1*: n=9 mice per group). **e** Relative MFI of 2-NBDG uptake in glyCAF determined by flow cytometry (n=5 mice per group). Source data are provided as a Source Data file.

Broz et al. Supplementary Fig. 4

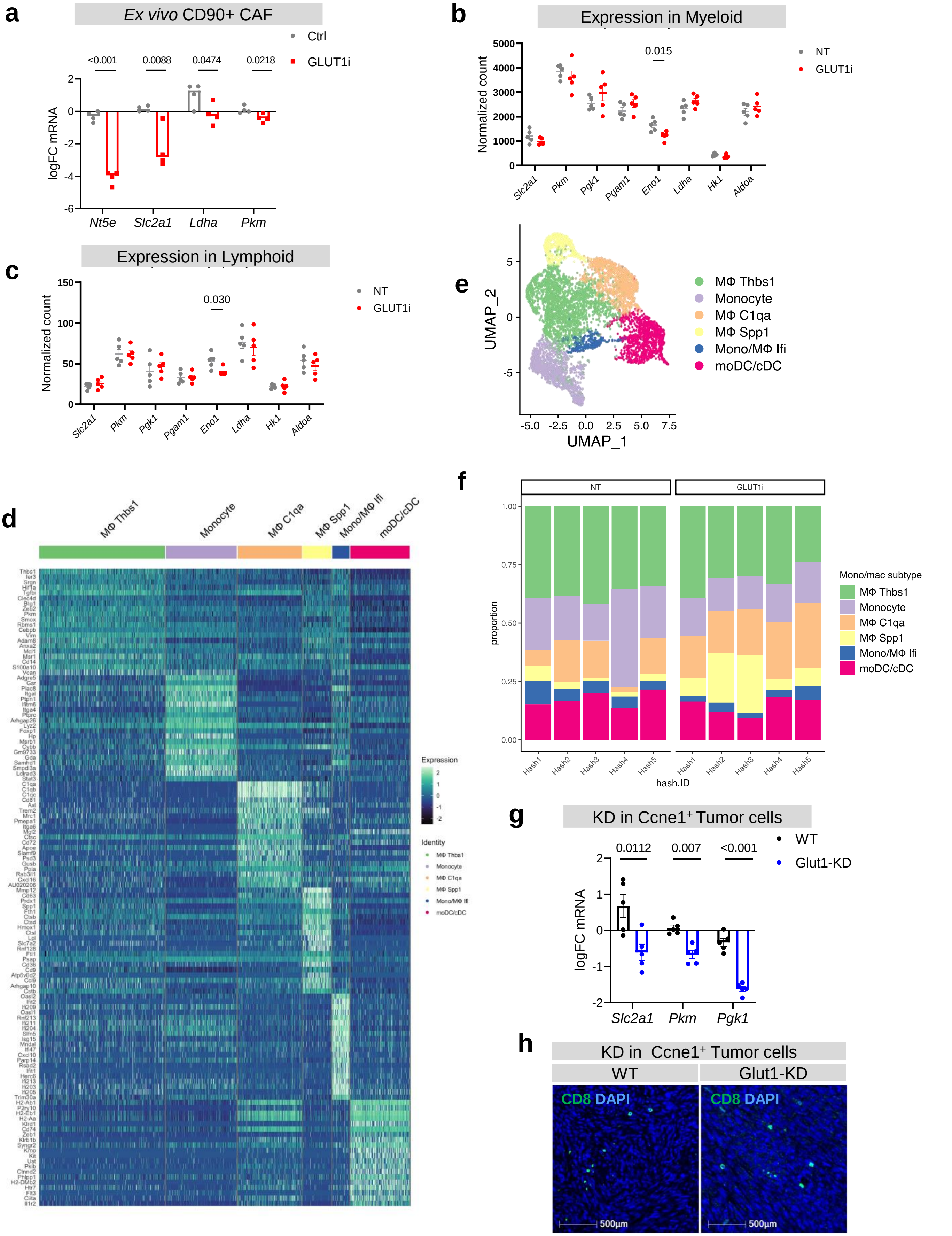

Supplementary Fig. 4: Effect GLUT1 inhibition on the TME

a qRT-PCR expression of *Slc2a1, Pgk1, and Ldha* in FACS-sorted dsRED+ tumor cells isolated from Ctrl or GLUT1i treated Ccne1+ tumors (n=4 mice per group). **b** Expression of the glycolytic genes in myeloid cells and **c** lymphoid cells from Ctrl (NT) or GLUT1i tumors. Dots represent individual mice. **d** Expression of top marker genes differentially expressed by the myeloid cell clusters. Dots represent individual mice. **e** t-SNE depicting several clusters of macrophages, monocytes, and dendritic cells (n=5 mice). **f** Proportions of myeloid cell phenotypes from NT and GLUT1i tumors, each hash/bar represents an individual mouse (n=5 mice). **g** qRT-PCR expression of *Slc2a1, Pkm,* and *Pgk1* in FACS-sorted $dsRED^{+}$ tumor cells isolated from WT or Glut1-KD $Ccne1^{+}$ tumors (n=5 mice). Data representative of two independent experiments. **h** Immunofluorescence staining of $CD8^{+}$ cells (green) infiltrating the tumor parenchyma of WT and Glut1-KD $Ccne1^{+}$ tumors. Source data are provided as a Source Data file.

Broz et al. Supplementary Fig. 5

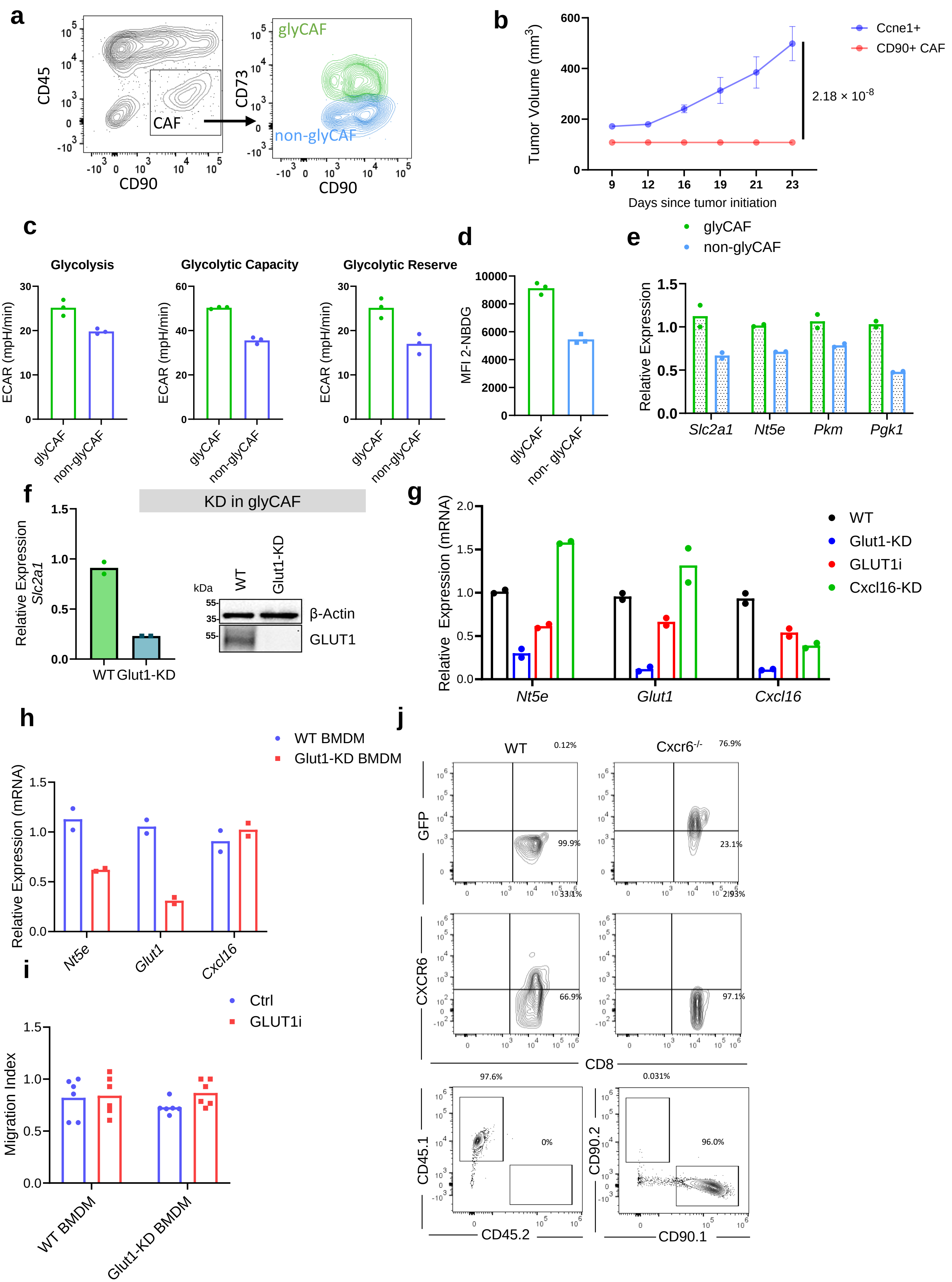

Supplementary Fig. 5: GLUT1 regulates CXCL16 expression in glyCAF

a Strategy for FACS sorting glyCAFs ($CD90^{+}$ $CD73^{+}$) and non-glyCAF ($CD90^{+}$ $CD73^{-}$) from the $Tumor^{-}$ $CD31^{-}$ fraction of $Ccne1^{+}$ tumor bearing mice for *in vitro* experiments. **b** Tumor growth curve for scaffolds implanted with 150k Ccne1 or CAF cells (n=3 mice). Two-way ANOVA with Tukey's multiple comparison was used to determine differences between groups. **c** Quantifications of glycolysis, glycolytic capacity, and glycolytic reserve from Seahorse metabolic profiling. Representative data from one experiment with n=3 technical replicates is shown out of three total experiments. **d** Median fluorescence intensity of 2-NBDG (FITC) after culturing glyCAF or non-glyCAF with 15uM of 2-NBDG for 30 minutes (n=3 technical replicates). **e** qRT-PCR gene expression glycolytic signature (*Slc2a1, Nt5e, Pkm, Pgk1,Hk2*) in cultured glyCAFs and non-glyCAFs after several passages *in vitro* (n=2 technical replicates). **f** mRNA expression of *Slc2a1* in cultured WT or Glut1-KD glyCAFs (n=2 technical replicates) (left) and Western blot of GLUT1 expression in WT or Glut1-KD glyCAFs(right). **g** mRNA expression of *Nt5e, Glut1, and Cxc16* in glyCAF treated with GLUT1i (BAY-876, 75um), Glut1-KD, or Cxcl16-KD glyCAF. Representative data from one experiment is shown out of three repetitions with n=2 technical replicates. **h** mRNA expression of Nt5e, Glut1, and Cxc16 in WT and Glut1-KD BMDM. Representative data from one experiment is shown out of two repetitions with n=2 technical replicates. **i** Migration index of T cells cultured in the presence of WT or Glut1-KD BMDM, with GLUT1i (BAY-876, 75uM). Representative data from one experiment is shown out of two repetitions with n=6 technical replicates. **j** Validation of CXCR6 GFP knock-in and congenic markers in CD8+ T cells purified from spleen. Source data are provided as a Source Data file.

Broz et al. Supplementary Fig. 6

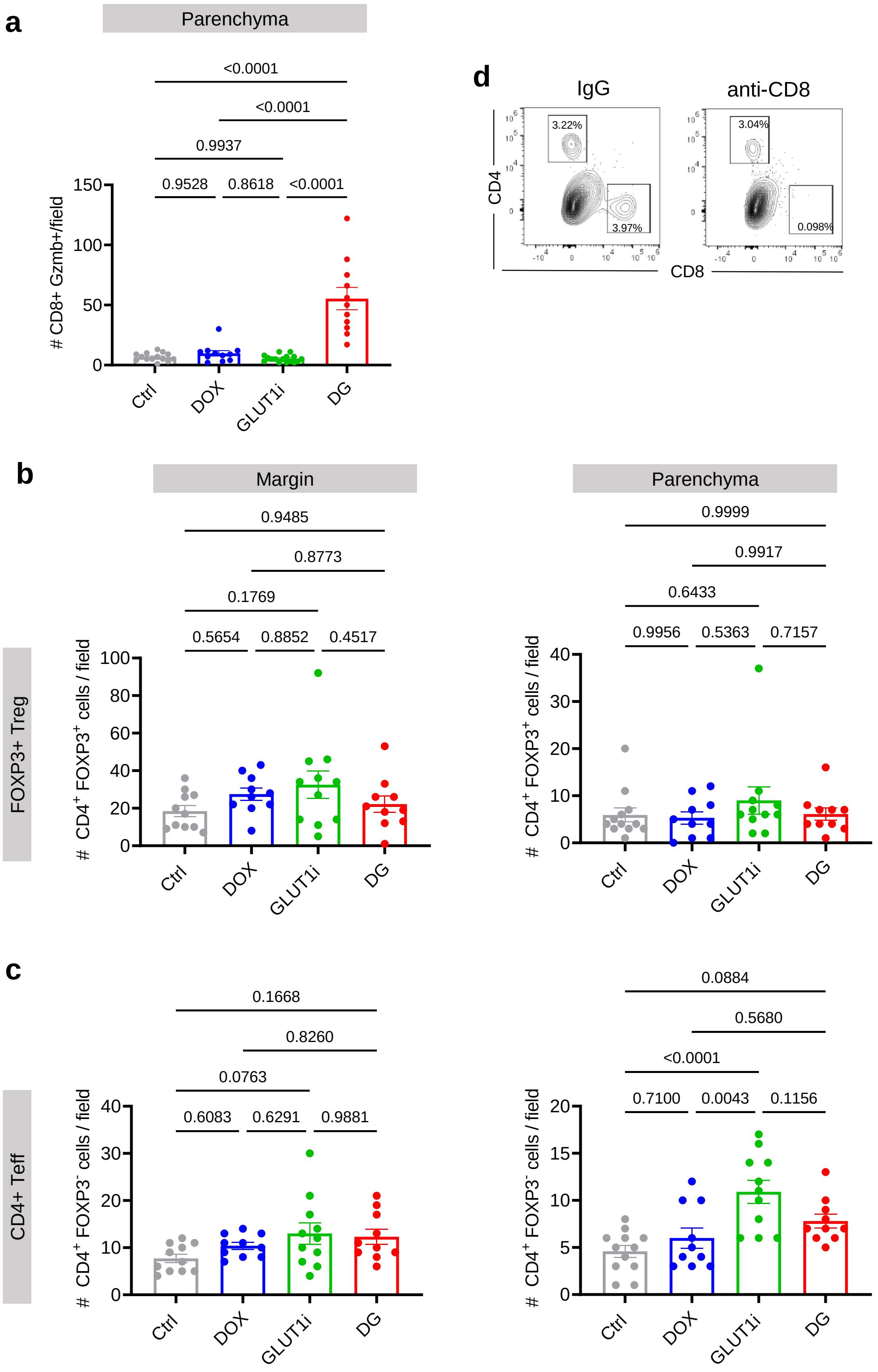

Supplementary Fig. 6: DOX/GLUT1i promotes CD8+ T cell cytotoxicity

a Multiplex immunohistochemistry quantification of $CD8^+$ $Gzmb^+$ cells in the tumor parenchyma (Ctrl: n= 14, DOX: n=16, GLUT1i: n=11, DG: n=11 ROIs from n=4 mice). **b** Multiplex immunohistochemistry quantification of $CD4^+$ $FOXP3^+$ cells localized in the tumor margin (Ctrl: n= 11, DOX: n=10, GLUT1i: n=11, DG: n=10 ROIs from n=4 mice) or parenchyma (Ctrl: n= 12, DOX: n=10, GLUT1i: n=11, DG: n=10 ROIs from n=4 mice). **c** Multiplex immunohistochemistry quantification of Teff $CD4^+$ $FOXP3^-$ cells localized in the tumor margin (Ctrl: n= 11, DOX: n=10, GLUT1i: n=11, DG: n=10 ROIs from n=4 mice). or parenchyma (Ctrl: n= 12, DOX: n=10, GLUT1i: n=11, DG: n=10 ROIs from n=4 mice). **d** Validation of $CD4^+$ and $CD8^+$ T cell depletion in the tumor mass 4 days following anti-CD4 or anti-CD8 neutralizing antibody administration as assessed by flow cytometry. Source data are provided as a Source Data file.
