# Supplementary material for: Metabolic targeting of cancer associated fibroblasts overcomes T-cell exclusion and chemoresistance in soft-tissue sarcomas
Source: Nat Commun. 2024 Mar 20;15:2498. doi: 10.1038/s41467-024-46504-4 (PMC10954767; doi:10.1038/s41467-024-46504-4)
Supplement: Supplementary file 3 — Reporting Summary [file 41467_2024_46504_MOESM3_ESM.pdf]

nature portfolio

Corresponding author(s): Jlenia Guarnerio

Last updated by author(s): 02/19/2024

Reporting Summary

Nature Portfolio wishes to improve the reproducibility of the work that we publish. This form provides structure for consistency and transparency in reporting. For further information on Nature Portfolio policies, see our Editorial Policies and the Editorial Policy Checklist.

Statistics

For all statistical analyses, confirm that the following items are present in the figure legend, table legend, main text, or Methods section.

n/a	Confirmed	
☐	☒	The exact sample size (*n*) for each experimental group/condition, given as a discrete number and unit of measurement
☐	☒	A statement on whether measurements were taken from distinct samples or whether the same sample was measured repeatedly
☐	☒	The statistical test(s) used AND whether they are one- or two-sided<br>*Only common tests should be described solely by name; describe more complex techniques in the Methods section.*
☐	☒	A description of all covariates tested
☐	☒	A description of any assumptions or corrections, such as tests of normality and adjustment for multiple comparisons
☒	☐	A full description of the statistical parameters including central tendency (e.g. means) or other basic estimates (e.g. regression coefficient) AND variation (e.g. standard deviation) or associated estimates of uncertainty (e.g. confidence intervals)
☐	☒	For null hypothesis testing, the test statistic (e.g. *F*, *t*, *r*) with confidence intervals, effect sizes, degrees of freedom and *P* value noted<br>*Give P values as exact values whenever suitable.*
☒	☐	For Bayesian analysis, information on the choice of priors and Markov chain Monte Carlo settings
☒	☐	For hierarchical and complex designs, identification of the appropriate level for tests and full reporting of outcomes
☒	☐	Estimates of effect sizes (e.g. Cohen's *d*, Pearson's *r*), indicating how they were calculated

Our web collection on statistics for biologists contains articles on many of the points above.

Software and code

Policy information about availability of computer code

Data collection	Flow Cytometry data were collected using the BD LRS II or Sony ID7000, cell sorting was performed with the BD Aria III. Western blot images were collected with the iBright imager (Thermo Fisher). RTqPCR data were generated with QuantStudio 3 (Applyed Biosystems). Single-cell partitioning was performed by using a 10X Chromium controller. RNA-seq data from TCGA Sarcoma PanCancer Atlas was queried in cBioPortal. H&E for the tumor sections were acquired by using a bright field microscope (Evos XL Core, Thermo Fisher). Immunofluorescence images were acquired using a fluorescence microscope (ECHO Revolve, ECHO). Seahorse Data was acquired using Agilent Seahorse XFp Analyzer.
Data analysis	All graphs were generated using Graphpad Prism v8 or R. T-test P-value was calculated either with Graphpad Prism v9. Flow Cytometry data were analyzed using FlowJo software v10. Multiplex immunofluorescence image analysis was performed in HALO HighPlexFL v3.6 (Indica Labs). Pathway enrichment analysis of RNA-seq data was performed with g:profiler. scRNAseq analyses used the following software: Cell Ranger (10X Genomics), Seurat, R v4.0, RStudio v1.3, ProjectTILs, and CellChat. Seahorse data was analyzed in Wave.

For manuscripts utilizing custom algorithms or software that are central to the research but not yet described in published literature, software must be made available to editors and reviewers. We strongly encourage code deposition in a community repository (e.g. GitHub). See the Nature Portfolio guidelines for submitting code & software for further information.

Data

Policy information about availability of data

All manuscripts must include a data availability statement. This statement should provide the following information, where applicable:

- Accession codes, unique identifiers, or web links for publicly available datasets
- A description of any restrictions on data availability
- For clinical datasets or third party data, please ensure that the statement adheres to our policy

Experimental data will be available from the corresponding author upon request. RNA sequencing data has been submitted for review to the NCBI Gene Expression Omnibus (GEO) - Accession number Super-Series: GSE237638. Sub-series: GSE237628 (human), GSE237636 (mouse).

Research involving human participants, their data, or biological material

Policy information about studies with human participants or human data. See also policy information about sex, gender (identity/presentation), and sexual orientation and race, ethnicity and racism.

Reporting on sex and gender	De-identified human UPS samples were used, therefore no population data are available except in the case of uterine leiomyosarcoma (LMS) which were collected from female subjects.
Reporting on race, ethnicity, or other socially relevant groupings	Not applicable.
Population characteristics	Not applicable.
Recruitment	Not applicable.
Ethics oversight	Human sarcomas for single-cell RNA sequencing were collected at Cedars Sinai Medical Center. Frozen tissue was provided by the Cedars Sinai BioBank and Research Pathology Resource, which received patient informed consent. FFPE blocks were provided by the Department of Pathology at Cedars Sinai. Because de-identified FFPE samples were used, this use does not qualify as human subject research, therefore no IRB approval was required.

Note that full information on the approval of the study protocol must also be provided in the manuscript.

Field-specific reporting

Please select the one below that is the best fit for your research. If you are not sure, read the appropriate sections before making your selection.

☒ Life sciences ☐ Behavioural & social sciences ☐ Ecological, evolutionary & environmental sciences

For a reference copy of the document with all sections, see nature.com/documents/nr-reporting-summary-flat.pdf

Life sciences study design

All studies must disclose on these points even when the disclosure is negative.

Sample size	No statistical analysis was used to predetermine sample size. Sample size was chosen based on the standard protocols in the field. All the independent biological replicates of the in vitro experiments are shown in the figures (single dots). For the mouse experiments, each mouse is shown as a dot in the presented charts. We used statistical analysis consistent with the sample size for each experiment and found sufficient statistical power with the sample sizes utilized in our study.
Data exclusions	No data exclusion.
Replication	All studies, unless otherwise indicated, were performed at least three times with the exception of the transwell migration experiment which was performed twice. For in vivo experiments, multiple mice were used to ensure reproducibility.
Randomization	In in vivo experiments animals were randomized to each experimental cohort.
Blinding	Tumor measurement and analysis was performed by operators blinded to the experimental groups. For the in vitro studies blinding was not relevant as all measures were quantified by standard cellular and biochemical assays. Key results were validated by 2 independent operators.

Reporting for specific materials, systems and methods

We require information from authors about some types of materials, experimental systems and methods used in many studies. Here, indicate whether each material, system or method listed is relevant to your study. If you are not sure if a list item applies to your research, read the appropriate section before selecting a response.

Materials & experimental systems

n/a	Involved in the study
☐	☒ Antibodies
☐	☒ Eukaryotic cell lines
☒	☐ Palaeontology and archaeology
☐	☒ Animals and other organisms
☒	☐ Clinical data
☒	☐ Dual use research of concern
☒	☐ Plants

Methods

n/a	Involved in the study
☒	☐ ChIP-seq
☐	☒ Flow cytometry
☒	☐ MRI-based neuroimaging

Antibodies

Antibodies used

Flow cytometry antibodies:
anti-CD45 PAC, BioLegend,#103126, clone 30-F11,Lot # B336453, 1:100
anti-CD8a APC-Cy7,BioLegend,#126620, clone YTS156.7.7,Lot # B267673, 1:100
anti-CD4 PE,BioLegend,#130310, clone H129.19,Lot # B216078, 1:100
anti-CD11b APC,BioLegend,#101212, clone M1/70,Lot # B368966, 1:100
anti-Ly6c PE,BioLegend,#128007, clone HK1.4,Lot # B298779, 1:100
anti-F4/80 FITC,BioLegend,#123107, clone BM8,Lot # B361743, 1:100
anti-NK1.1 APC-Cy7,BioLegend,#108723, clone PK136,Lot # B261757, 1:100
anti-CD90 FITC,BioLegend,#105306, clone 30-H12,Lot # B377019, 1:100
anti-CD73 APC,BioLegend,#127210, clone TY/11.8,Lot # B355340, 1:100
anti-CD31 PE,eBioscience,#12-0311-82, clone 390,Lot # 2270770, 1:100
anti-GranzymeB APC,BioLegend,#396408, clone QA18A29,Lot # B362691, 1:100
anti-PD-1 APC, BioLegend, #109111, clone RMP1-30, 1:100
anti-GLUT1,Novus Biologicals,#NB300-666,Lot #Fgi.72, 1:100
Goat anti-Rabbit IgG (H+L) Alexa Fluor 647,Invitrogen,#A21245,Lot # 2299231, , 1:100
anti-CXCL16, Bioss, #BS-1441R, , 1:50
anti-CXCR6 APC, BioLegend, #151105, clone #SA051D1, , 1:100

Western Blot antibodies:
anti-GLUT1Novus Biologicals#NB300-666,Lot #Fgi.72, 1:1000
anti-Beta ActinBethyl#A300-485A, 1:1000

Multiplex Immunofluorescence Antibodies:
anti-CD8,ebioscience#14-0808-82,4SM15,Lot # 2470230, 1:200
anti-CD4,R&D#MAB554, GK1.5,Lot # FHP0422121, 1:100
anti-CD90,Sino Biological#50461-T44,Lot # HDA11AU1510, 1:200
anti-CD73,Sino Biological#50231-T56,Lot # HD11JL2672, 1:200
anti-Granzyme B,ebioscience,#14-8822-82,16G6,Lot # 2265267, 1:200
anti-CD90.1, BioLegend, #202501, OX-7, 1:200
anti-CD45.1, Invitrogen, #14-0453-82, A20, 1:200
Goat Anti-Rat IgG H&L (HRP polymer),Abcam,#214882,Lot # GR3432519-1, pre-diluted
Goat Anti-Rabbit IgG H&L (HRP polymer),Abcam,#214880,Lot # 1038869-1, pre-diluted
In vivo antibodies:
InVivoMAb anti-mouse CD8α,BioXcell,#BE0061,2.43, 100ug
InVivoMAb rat IgG2b isotype control, anti-keyhole limpet hemocyanin,BioXcell,#BE0090,LTF-2, 100ug

Validation

All primary antibodies were confirmed on the species and application through the validation statement on the manufacturer's website and their use in the literature. We are providing here the list of antibodies used.

anti-CD45 PAC, BioLegend,#103126, clone 30-F11,Lot # B336453, 1:100, validated by the company (https://www.biolegend.com/fr-ch/clone-search/pacific-blue-anti-mouse-cd45-antibody-3102?GroupID=BLG6841) and by users (cited 184 times)
anti-CD8a APC-Cy7,BioLegend,#126620, clone YTS156.7.7,Lot # B267673, 1:100, validated by the company https://www.biolegend.com/fr-ch/products/apc-cyanine7-anti-mouse-cd8b-antibody-10021) and by users (cited 15 times)
anti-CD4 PE,BioLegend,#130310, clone H129.19,Lot # B216078, 1:100, validated by the company (https://www.biolegend.com/fr-ch/products/pe-anti-mouse-cd4-antibody-5488) and by users (cited 7 times)
anti-CD11b APC,BioLegend,#101212, clone M1/70,Lot # B368966, 1:100, validated by the company (https://www.biolegend.com/fr-ch/products/apc-anti-mouse-human-cd11b-antibody-345) and by users (cited 356 times)
anti-Ly6c PE,BioLegend,#128007, clone HK1.4,Lot # B298779, 1:100 , validated by the company (https://www.biolegend.com/fr-ch/products/pe-anti-mouse-ly-6c-antibody-4904) and by users (cited 78 times)
anti-F4/80 FITC,BioLegend,#123107, clone BM8,Lot # B361743, 1:100 , validated by the company (https://www.biolegend.com/fr-ch/products/fitc-anti-mouse-f4-80-antibody-4067) and by users (cited 207 times)
anti-NK1.1 APC-Cy7,BioLegend,#108723, clone PK136,Lot # B261757, 1:100, validated by the company (https://www.biolegend.com/fr-ch/products/apc-cyanine7-anti-mouse-nk-1-1-antibody-4002) and by users (cited 50 times)

anti-CD90.2 FITC,BioLegend,#105306, clone 30-H12,Lot # B377019, 1:100, validated by the company (https://www.biolegend.com/fr-ch/products/fitc-anti-mouse-cd90-2-thy1-2-antibody-104) and by users (cited 31 times)
anti-CD73 APC,BioLegend,#127210, clone TY/11.8,Lot # B355340, 1:100, validated by the company (https://www.biolegend.com/fr-ch/products/apc-anti-mouse-cd73-antibody-7893) and by users (cited 14 times)
anti-CD31 PE,eBioscience,#12-0311-82, clone 390,Lot # 2270770, 1:100, validated by the company (https://www.thermofisher.com/antibody/product/CD31-PECAM-1-Antibody-clone-390-Monoclonal/12-0311-82) and by users (cited 134 times)
anti-GranzymeB APC, BioLegend,#396408, clone QA18A29,Lot # B362691, 1:100, validated by the company (https://www.biolegend.com/fr-ch/search-results/apc-anti-human-mouse-granzyme-b-recombinant-antibody-17568) and by users (cited 1 time)
anti-PD-1 APC, BioLegend, #109111, clone RMP1-30, 1:100, validated by the company (https://www.biolegend.com/nl-nl/products/apc-anti-mouse-cd279-pd-1-antibody-66728) and by users (cited 21 times)
anti-GLUT1,Novus Biologicals,#NB300-666,Lot #Fgi.72, 1:100, validated by the company (https://www.novusbio.com/products/glut1-antibody_nb300-666) and by users (cited 29 times)
Goat anti-Rabbit IgG (H+L) Alexa Fluor 647,Invitrogen,#A21245,Lot # 2299231, 1:100, validated by the company (https://www.thermofisher.com/antibody/product/Goat-anti-Rabbit-IgG-H-L-Highly-Cross-Adsorbed-Secondary-Antibody-Polyclonal/A-21245) and by users (cited 1701 times)
anti-CXCL16, Bioss, #BS-1441R, 1:50, validated by the company (https://www.biossusa.com/products/bs-1441r) and by users (cited 1 time)
anti-CXCR6 APC, BioLegend, #151105, clone #SA051D1, 1:100, validated by the company (hhttps://www.biolegend.com/fr-ch/products/apc-anti-mouse-cd186-cxcr6-antibody-13065) and by users (cited 5 times)

Western Blot antibodies:
anti-GLUT1,Novus Biologicals,#NB300-666,Lot #Fgi.72, 1:1000, validated by the company (https://www.novusbio.com/products/glut1-antibody_nb300-666) and by users (cited 29 times)
anti-Beta ActinBethyl#A300-485A, 1:1000, validated by the company (https://www.fortislife.com/products/primary-antibodies/rabbit-anti-cytoskeletal-actin-antibody/BETHYL-A300-485) and by users (cited 27 times)

Multiplex Immunofluorescence Antibodies:
anti-CD8,ebioscience#14-0808-824SM15,Lot # 2470230, 1:200, validated by the company (https://www.thermofisher.com/antibody/product/CD8a-Antibody-clone-4SM15-Monoclonal/14-0808-82) and by users (cited 106 times)
anti-CD4,R&D#MAB554, GK1.5,Lot # FHP0422121, 1:100, validated by the company (https://www.rndsystems.com/products/mouse-cd4-antibody-gk15_mab554) and by users (cited 14 times)
anti-CD90,Sino Biological#50461-T44,Lot # HDA11AU1510, 1:200, validated by the company (https://www.sinobiological.com/antibodies/mouse-thy1-cd90-50461-t44) and by users (cited 2 times)
anti-CD73,Sino Biological#50231-T56,Lot # HD11JL2672, 1:200, validated by the company (https://www.sinobiological.com/antibodies/mouse-cd73-50231-t56).
anti-Granzyme B,ebioscience,#14-8822-82,16G6,Lot # 2265267, 1:200, validated by the company (https://www.thermofisher.com/antibody/product/Granzyme-B-Antibody-clone-16G6-Monoclonal/14-8822-82) and by users (cited 22 times)
anti-CD90.1, BioLegend, #202501, OX-7, 1:200, validated by the company (https://www.biolegend.com/fr-ch/products/purified-anti-rat-cd90-mouse-cd90-1-thy-1-1-antibody-2411?GroupID=BLG10566) and by users (cited 15 times)
anti-CD45.1, Invitrogen, #14-0453-82, A20, 1:200, validated by the company (https://www.thermofisher.com/antibody/product/CD45-1-Antibody-clone-A20-Monoclonal/14-0453-82) and by users (cited 134 times)
Goat Anti-Rat IgG H&L (HRP polymer),Abcam,#214882,Lot # GR3432519-1, pre-diluted, validated by the company (https://www.abcam.com/products/secondary-antibodies/goat-rat-igg-hl-hrp-polymer-ab214882.html) and by users (cited 6 times)
Goat Anti-Rabbit IgG H&L (HRP polymer),Abcam,#214880,Lot # 1038869-1, pre-diluted, validated by the company (https://www.abcam.com/products/secondary-antibodies/goat-rabbit-igg-hl-hrp-polymer-ab214880.html) and by users (cited 37 times)

In vivo antibodies:
InVivoMAb anti-mouse CD8α,BioXcell,#BE0061,2.43, 100ug, validated by the company (https://bioxcell.com/invivomab-anti-mouse-cd8-alpha-be0061#tab_references) and by users (cited 17 times)
InVivoMAb rat IgG2b isotype control, anti-keyhole limpet hemocyanin,BioXcell,#BE0090,LTF-2, 100ug, validated by the company (https://bioxcell.com/invivomab-rat-igg2b-isotype-control-anti-keyhole-limpet-hemocyanin-be0090) and by users (cited 15 times)

Eukaryotic cell lines

Policy information about cell lines and Sex and Gender in Research

Cell line source(s)	HEK 293T cells were purchased from ATCC.
Authentication	No other independent authentication was performed.
Mycoplasma contamination	HEK 293T cell line were tested negative for mycoplasma contamination.
Commonly misidentified lines (See ICLAC register)	None of these cell lines were used in this study.

Animals and other research organisms

Policy information about studies involving animals; ARRIVE guidelines recommended for reporting animal research, and Sex and Gender in Research

Laboratory animals	Wild type mice C57BL/6 (#000664) and p53KO mice (#002101) were purchased from The Jackson Laboratory. Cxcr6-/- OT-1 and Cxcr6wt/wt OT-1 mice were a gift from Amanda Lund in agreement with NYU Grossman School of Medicine. Briefly, these mice were generated by crossing B6.SJL-PtprcaPepcb/BoyJ (CD45.1), C57BL/6-Tg(TcraTcrb)1100Mjb/J (OT-1), B6.129P2-Cxcr6tm1Litt/J

	(CXCR6-/-)66, B6.PL-Thy1a/CyJ (CD90.1), which were purchased from Jackson Laboratory. Males and Females (12 weeks old to 6 months old) were maintained and used for breeding purposes.
Wild animals	This study did not involve wild animals.
Reporting on sex	Syngeneic modeling of Ccne1+ and Vgll3+ tumors utilized mesenchymal stem cells isolated from female mice, thus sex-matched females were used as tumor recipients for all experiments.
Field-collected samples	This study did not involve field collected samples.
Ethics oversight	Animal experiments were performed in accordance with the guidelines of Cedars-Sinai Medical Center Institutional Animal Care and Use Committee.

Note that full information on the approval of the study protocol must also be provided in the manuscript.

Flow Cytometry

Plots

Confirm that:

☒ The axis labels state the marker and fluorochrome used (e.g. CD4-FITC).

☒ The axis scales are clearly visible. Include numbers along axes only for bottom left plot of group (a 'group' is an analysis of identical markers).

☒ All plots are contour plots with outliers or pseudocolor plots.

☒ A numerical value for number of cells or percentage (with statistics) is provided.

Methodology

Sample preparation	Tumors were dissociated by enzymatic digestion with the OctoMacs (Miltenyi) to single cell suspension. Cells were filtered twice through 70 µm filters. Red blood cells were lysed with ACK solution (Gibco). After ACK, cells were washed twice with PBS, and then stained with the fluorophore-conjugated antibodies for 15 minutes at room temperature. The excess of unbound antibodies was washed out before acquisition in flow cytometry.
Instrument	BD FACSAria, Sony ID7000, and BD LSR II.
Software	Flow cytometry data was collected with BD FACS Diva 8.0.3 or Sony ID7000 software and analyzed with FlowJo v10.
Cell population abundance	N/A
Gating strategy	Forward scatter vs. side scatter plot was used to separate cell events from debris. Dead cells were removed from the analysis by gating on cells negative for viability dye. Non-stained negative controls were used in the experiments to define gating.

☒ Tick this box to confirm that a figure exemplifying the gating strategy is provided in the Supplementary Information.
